# Shaping experimental orthopaedics

**DOI:** 10.1186/s40634-023-00658-0

**Published:** 2023-09-24

**Authors:** Henning Madry

**Affiliations:** Institute of Experimental Orthopaedics, Saarland University, Kirrberger Straße, Building 37, 66421 Homburg, Saar Germany

*The library will endure; it is the universe. As for us, everything has not been written; we are not turning into phantoms. We walk the corridors, searching the shelves and rearranging them, looking for lines of meaning amid leagues of cacophony and incoherence, reading the history of the past and our future, collecting our thoughts and collecting the thoughts of others, and every so often glimpsing mirrors, in which we may recognize creatures of the information*.

The Library of Babel

Jorge Luis Borges (1899—1986)

Nearly 10 years ago, the Journal of Experimental Orthopaedics was launched by the European Society of Sports Traumatology, Knee Surgery & Arthroscopy (ESSKA). The vision behind its establishment was to create an open access journal that complemented the esteemed Knee Surgery, Sports Traumatology, Arthroscopy (KSSTA) journal with a journal focusing on basic orthopaedic research while maintaining a strong surgical background [13]. The structure of open access was selected to increase the visibility and impact of the published research, and more and more institutions have agreed to provide fundamental financing for such a publishing model. Yet, this also raised the theoretical question whether any kind of submission would be accepted for publication, just to earn money, as it is the case for the so-called predatory journals. Therefore, my vision as Founding Editor-in-Chief of the Journal of Experimental Orthopaedics was to establish a journal solely focusing on scientific excellence, while also helping our orthopaedic community to publish their work based on quality, trustworthiness and merit, by generating a rigorous yet objective peer review process. The strong affiliation of the journal to the influential ESSKA, reflecting the high values of this exceptional community of orthopaedic surgeons, clinicians and scientists in Europe and worldwide provided a strong tailwind supporting the Journal.

Already in the past 10 years since its launch, the Journal of Experimental Orthopaedics has influenced the trajectory and shape of the global landscape of orthopaedic research. Since the publication of its first article, 638 articles have been published, attesting to its impact. Part of this success is that the Journal has been supported and enriched by highly commendable initiatives of the many committees and groups within and outside of ESSKA. Good examples are the work of the ESSKA Basic Science Committee on tendinopathy [1, 2], the ESSKA Paediatric Anterior Cruciate Ligament (ACL) Initiative registry [16], addressing ACL reconstruction with open growth plates [18], recommendations from a Society for Orthopaedic-Traumatological Sports Medicine (GOTS) expert meeting on treatment of muscle injuries [10], and the ESSKA guidelines for resuming elective surgery after the COVID-19 pandemic [15]. With such remarkable publications, the Journal not only provides a vehicle for publication and dissemination, but also stimulates collaborations within our community.

The vast range of themes covered ranges from basic science topics like aggrecan, the major proteoglycan in the articular cartilage [17], biomaterials for osteochondral regeneration [12] to the kinematics of a medial meniscus replacement [19]. Without doubt, evolution of a journal involves changes. Over the past years, clinical related topics gained more place, such as technical notes [9], case reports [14], case series [5, 8], clinical trials [6], and many state-of-the-art clinical or review papers like on ACL reconstruction [3, 4, 20] or the highly accessed guide on management of an infected total knee replacement [21]. Another noteworthy example of the broad scope of the Journal is the fine publication on “Tips and tricks for building a good paper”, written by the Editors of the three most prestigious clinical journals in our field [11]. These achievements and hard work of all persons involved culminated in the first-ever journal impact factor of 1.8 that the Journal of Experimental Orthopaedics just recently received.

What lies ahead? Artificial intelligence is a potentially powerful tool but likewise a debated topic in the academic and publishing community [7]. In his fictional short story masterpiece containing the above quote, the Argentine author Jorge Luis Borges (1899–1986) envisioned a library containing a universe of books whose contents are randomly constituted, solely based on all possible combinations of 22 letters, the period, the comma, and space. Of course, the books of Borges’ library must also contain certain true meanings, while artificial intelligence combs through the huge amounts of already available writings, arranging the text with elaborate algorithms. It is thus intriguing to contemplate whether such artificial intelligence may replace the creativity, discernment and sharpness of the human mind. What would happen if artificial intelligence generates scientific papers, or parts thereof like an Introduction or Discussion, or even an entire Narrative Review? We need to find answers and provide guidance, most importantly to guarantee that “*VERITAS*”, the truth, remains the ultimate goal of all science conducted and published.

I am convinced that the Journal of Experimental Orthopaedics will continue to represent a trustworthy and indispensable resource for our community of orthopaedic researchers and clinicians, providing a basis for the high-quality care of the patients that we wish to serve with our work.

Sincerely,

Henning Madry

Founding Editor-in-Chief

Journal of Experimental Orthopaedics

## Data Availability

Not applicable.
